# Corrosion of Carbon Steel in an Arsenic Trioxide Reduction Atmosphere Using Carbonaceous Materials for Elemental Arsenic Production

**DOI:** 10.3390/ma19020336

**Published:** 2026-01-14

**Authors:** Xiao Long, Wenbo Luo, Kai Zheng, Bo Feng, Xiang Li, Jierui Li

**Affiliations:** 1 School of Materials and Energy Engineering, Guizhou Institute of Technology, Guiyang 550025, China; kaizhengshu@163.com (K.Z.); lixiang8656@163.com (X.L.); 18798005157@163.com (J.L.); 2 College of Resources and Environment, Zunyi Normal University, Zunyi 563006, China; dabosucceed@163.com

**Keywords:** elemental arsenic, reduction, reactor corrosion, carbon particles, Fe–As–C system

## Abstract

Elemental arsenic (As) is essential for diverse industrial applications. Most elemental As in China is produced by reducing gaseous arsenic trioxide (As_2_O_3_) with carbonaceous materials in steel reactors. This study aimed to extend the reactor lifespan through corrosion experiments and analysis. In this study, corroded regions of steel reactors were inspected after each production batch, and the corrosion process was examined. X-ray diffraction (XRD) was used to identify the major corrosion products, X-ray fluorescence (XRF) was used to measure the composition of corroded area, scanning electron microscopy (SEM) and energy-dispersive spectroscopy (EDS) were used to inspect the features and elemental distributions of the corroded steel-plate cross-sections. The results revealed that the steel wall near the charcoal zone exhibited the highest corrosion rate. Tin (Sn), selenium (Se), and antimony (Sb) did not promote the corrosion process, whereas carbon (C) accelerated it by forming an Fe–As–C system at the grain boundaries of the steel matrix, characterized by a low melting temperature. The important source of C responsible for initiating corrosion was solid-state C particles originating from reused materials from previous batches. Additionally, owing to the high processing temperature, oxygen (O) was transferred to the inner side of the steel wall before the dramatical corrosion of the matrix by elemental As and C. Results of this study provide references to increase the lifespan of steel reactors for elemental As production.

## 1. Introduction

Elemental arsenic (As) is an essential material used in the alloy production, medical (such as chemotherapeutic drugs), chemical (such as insecticides and preservatives), and semiconductor manufacturing fields [1,2,3]. As one of the major raw materials used in the production of third-generation semiconductors, the manufacture and purification of elemental As is currently the focus of related research and is of considerable importance to the smooth running of subsequent procedures [4,5,6,7].

**Figure 1:**
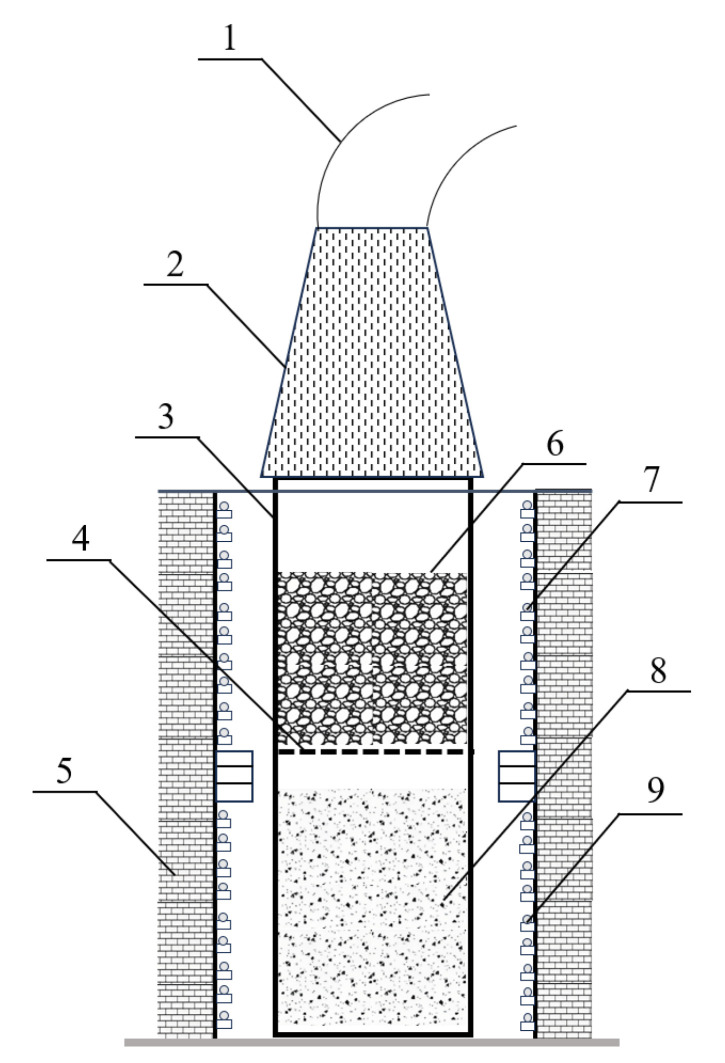
Schematic diagram of the equipment used for elemental arsenic manufacturing: 1: flue; 2: condenser; 3: steel reactor; 4: grate; 5: refractory brick; 6: charcoal. 7: upper heating zone; 8: arsenic trioxide; and 9: lower heating zone.

Numerous achievements have been reported on producing elemental As using various kinds of waste from non-ferrous metal production [8,9,10,11]. In China, most elemental As is produced by the reduction of gaseous arsenic trioxide (As_2_O_3_) using carbonaceous materials [12]. The entire reduction process is conducted in a cylindrical steel reactor (Figure 1). Solid raw materials containing As_2_O_3_ for reduction are loaded in the lower part of the reactor, and carbonaceous materials are loaded in the upper part. After sealing and placing the reactor in an induction furnace and then heating it to the reduction temperature at specific velocities, As_2_O_3_ is vaporized and reacts with the preheated carbonaceous materials to form gaseous elemental As. Finally, α-phase As can be obtained by condensing the generated As vapor at specific temperatures. During the manufacturing of elemental As, owing to the high processing temperature and complicated atmosphere, the corrosion rate and behavior of steel reactors are rapid and complex. Consequently, the average lifespan of steel reactors is extremely short (five to eight production batches) [12], which poses serious environmental pollution risks because of the increased As-containing steel scrap generation during reactor maintenance or replacement [13,14,15,16].

To increase the lifespan of reactors, special steels such as stainless steels and heat-resistant steels have been used. Corrosion-resistant coatings have also been tentatively applied. However, commercial applications have shown that these methods do not increase the lifespan of steel reactors but can increase the equipment cost or potentially affect the product quality. It is generally acknowledged that the corrosion of steel reactors in elemental As manufacturing is facilitated by the formation of Fe–As compounds with low melting temperatures. However, the corrosion process and the effects of other components, such as O, C, Sn, Se, and Sb, on the corrosion process remain unclear. As simulating the reduction of gaseous As_2_O_3_ in the laboratory can be difficult and involve safety risks (high volatility and high toxicity), few studies have been reported on the thermodynamics and kinetics information of the corrosion process. Elucidating the corrosion process in detail can help in addressing the corrosion problem and enhancing both the product quality and reactor lifespan.

Accordingly, corrosion experiments on steel reactors were conducted, and the corrosion process and the effects of other components (O, C, Sn, Se, and Sb) on the corrosion process were examined and discussed in this study. Results of this study provide references to increase the lifespan of steel reactors for elemental As production.

## 2. Materials and Methods

### 2.1. Sample Acquisition

**Figure 2:**
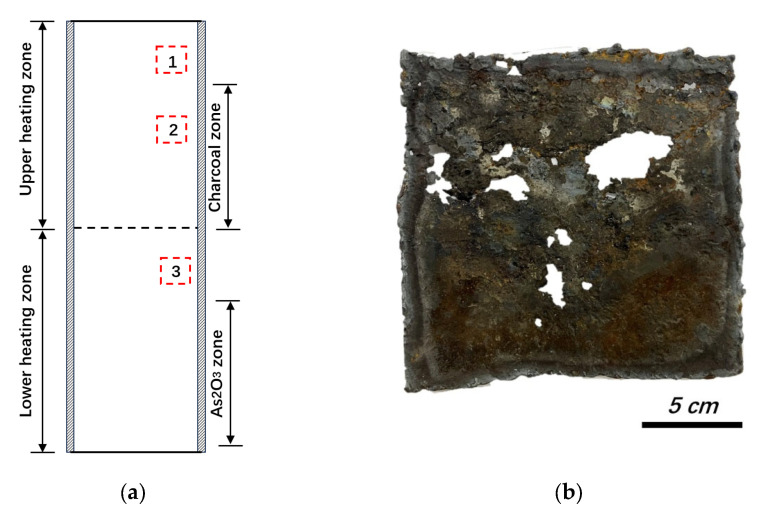
(**a**) Position for sampling (labeled with red dashed line); (**b**) typical appearance of a sample from Position 2 (with corrosion perforations).

To reveal the corrosion behavior on the inner side of steel reactors, experiments were conducted using As_2_O_3_ (purity > 98%) and charcoal as raw materials. The reactor (580-mm diameter and 2150-mm length) was fabricated from conventional C steel of 8 mm thickness. The composition of the steel is listed in Table 1. As the manufacturing process is intermittent, low-purity products, dust, and unspent charcoal from previous batches were also adopted as raw materials. For each batch, the upper charcoal zone was first heated to 720 °C, after which the lower As_2_O_3_ zone was heated to 700 °C at a rate of 2–3 °C per minute to vaporize the As_2_O_3_. The main reaction is As_2_O_3_ (g) + 3C (s) = 2As (g) + 3CO (g). After the reaction was complete, the reactor was cooled to room temperature (25 to 35 °C), and the corrosion products from the inner side of the steel wall were harvested by scraping. When corrosion perforation occurred, samples (steel plates of 150 mm length and 150 mm width) were cut and collected from the steel wall, the sampling location of which is shown in Figure 2 (labeled with a red dashed line).

### 2.2. Measurements and Analysis

To determine the corrosion rate of the steel wall, an ultrasonic thickness gauge (Olympus Corporation, 39DL Plus, Tokyo, Japan) was used to measure the thickness of the steel wall after each production batch. The measurement positions were chosen at the charcoal zone, lower heating zone, and upper part of the reactor (near the center of Positions 1, 2, and 3 in Figure 2). X-ray diffraction (XRD, RIGAKU Corporation, MiniFlex, Tokyo, Japan) was used to identify the major corrosion products and X-ray fluorescence (XRF, RIGAKU Corporation, ZSX Primus III+, Tokyo, Japan) was used to measure their compositions after grinding the corroded parts into fine powders (<75 μm). Scanning electron microscopy (SEM, FEI Company, Nova Nanosem 450, Hillsboro, OR, USA) and energy-dispersive spectroscopy (EDS) were used to inspect the features and elemental distributions of the corroded steel-plate cross-sections. Small samples for SEM-EDS inspection were first mounted on a resin, polished with alumina slurry, and then sputter-coated with gold (Au).

## 3. Results and Discussion

### 3.1. Corrosion Rate

**Figure 3:**
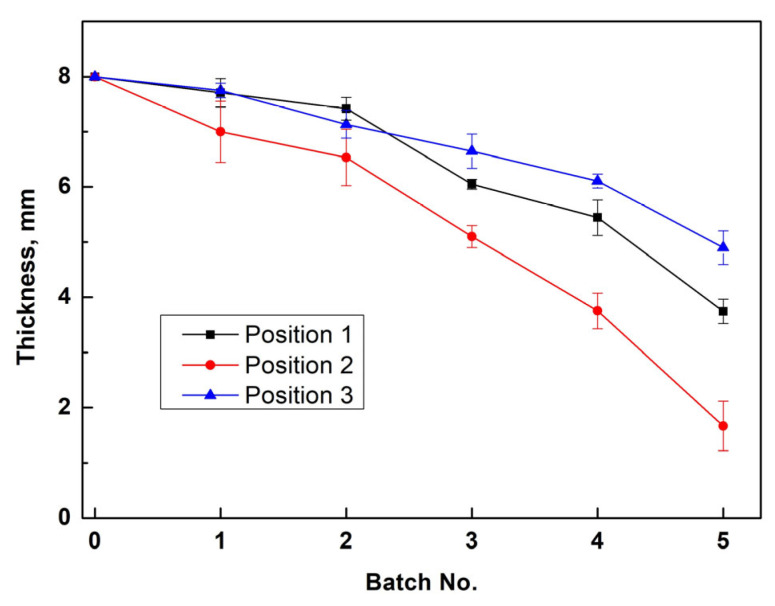
Remaining thickness of the steel wall at different positions after different production batches (error bars give standard deviations).

The measured plate thicknesses are shown in Figure 3. Six production batches were run in the steel reactor. As corrosion perforations occurred near Position 2 during the sixth production batch, the thickness data for batch 6 were not plotted. Based on the thickness data, it can be concluded that the steel wall near Position 2 (charcoal zone) was more likely to be corroded in the first production batch. The corrosion rates of the steel wall at Positions 1 and 3 were lower and similar prior to the third production batch. The reason for the more rapid steel-wall corrosion at Position 2 (charcoal zone) will be discussed in Section 3.3.

### 3.2. Corrosion Products

Gaseous elemental As can be expected to react with steel to form metallic compounds with low melting temperatures, resulting in corrosion. However, other impurities within raw materials, such as Sn, Sb, and Se, have also been considered to affect the corrosion process but this has not yet been proven in commercial practice. In this study, XRD and XRF were used to identify the corrosion products and elementary compositions of the acquired samples.

**Figure 4:**
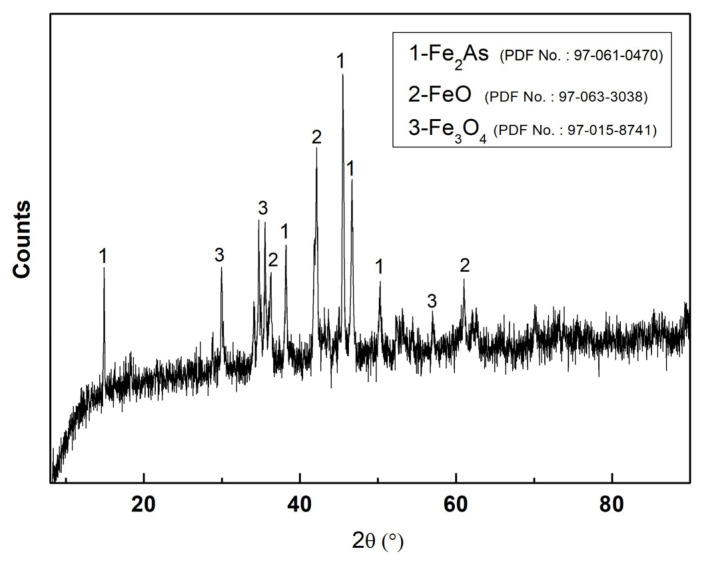
Typical XRD pattern of a corroded sample obtained from Position 2.

Table 2 lists the typical XRF results of the corroded samples obtained from the different positions, indicates that in addition to Fe and As, the other detected components, Mn and Si, should be originated from the steel material. Sn or Sb were not detected and only a trace quantity of Se was evident (which should have no impact on the corrosion process, the detection limits of Sn, Sb, and Se using XRF are 10 ppm). This is further proven in the next section. The XRD results in Figure 4 indicate that the main crystal phases formed in the corrosion perforation area (Position 2) were Fe_2_As, FeO, and Fe_3_O_4_, suggesting that oxidation processes from outside the steel reactor could also have had an impact on the corrosion perforations.

### 3.3. Effects of Carbon and Oxygen on Corrosion Process

**Figure 5:**
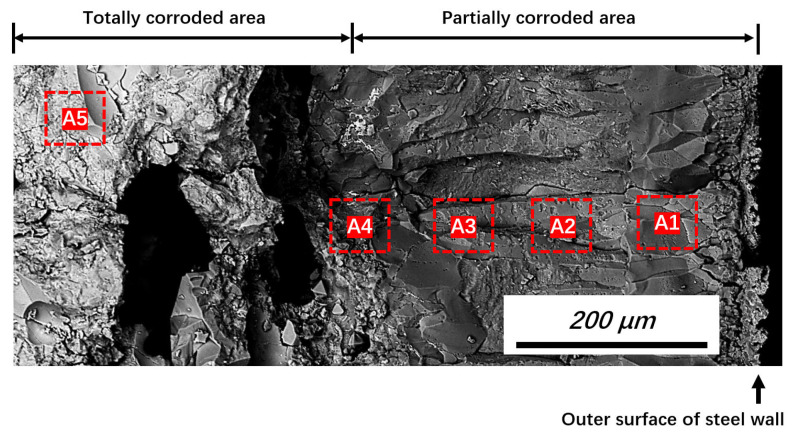
Typical fracture appearance obtained from Position 2 (backscattered electron image).

To reveal the corrosion process of the steel reactors, cross-sections of the corroded steel wall were inspected using SEM-EDS. Figure 5 shows the typical appearance of a fracture in the steel wall near Position 2. Two typical areas—that is, totally and partially corroded—were observed. Solidification segregation and reactions caused abrupt compositional changes in micro-areas. Consequently, instead of point or line scanning, EDS area scanning was adopted to measure the average composition and its change tendency within different parts of the totally and partially corroded areas.

The positions for the measurements are shown in Figure 5 (red dashed lines), with the measurement positions (A1–A5) representing different distances from the outer surface of the steel wall. To ensure data accuracy, five measurements were performed at each distance (different longitudinal locations in Figure 5), and the average value was recorded.

**Figure 6:**
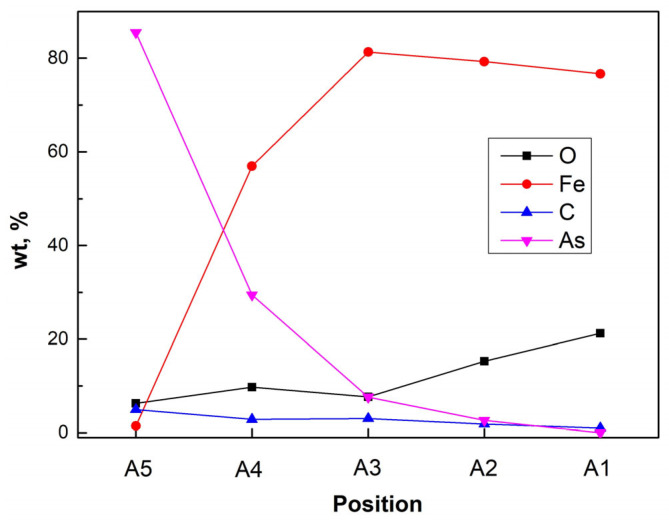
Composition changes of different areas of the cross-section.

The main compositions measured are shown in Figure 6. The results indicate that during the production process, oxygen (O) was transferred to the inner side of the steel wall, which promoted the corrosion process. In addition to O, C showed a similar change tendency as As, which increased from areas A1 to A5. (from 1.02 wt% to 4.96 wt%). Although the C content based on EDS measurements was only semi-quantitative, the existence and change tendency could be confirmed. Adding C decreased the eutectic temperature of the Fe–As–C system (the eutectic temperature decreased to 810 °C with 20.4 mol% of As and 4.1 mol% of C) [17,18]. This result indicates that C promoted the corrosion process to some degree. The difference between eutectic temperature and processing temperature can reflect the reactivity of phases or substances. Closer to the eutectic temperature (melting temperature in heating process) means lower activation energies of reactions are required to break the old bonds and lower activation energies for diffusion. Thus, a lower eutectic temperature with the addition of C can promote the corrosion process, even if no liquid phases are formed during processing. As rare references have been reported on the As-Fe-C based system, research on the behavior of the C in As–Fe–C systems, especially its thermodynamic and kinetic properties, should be conducted in the future. In the corrosion zone, Sn, Sb, and Se were practically not detected, which further confirms the previously made conclusion about the absence of their influence on the corrosion process.

**Figure 7:**
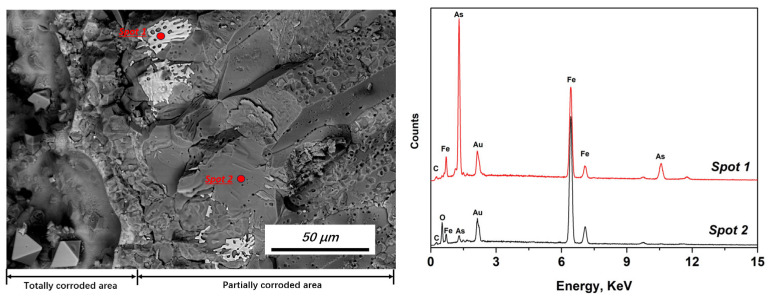
Appearance of a typical fracture surface at the interface between the totally and partially corroded areas (backscattered electron image) and EDS pattern of the corresponding spot measurements.

The average composition change (EDS area scanning) can be used to explain and predict the corrosion tendency; however, it cannot reveal the initial corrosion process. Thus, a typical area showing the initial corrosion features was examined. Figure 7 shows the interfacial area between the completely and partially corroded areas. Although most of the initial corrosion products peeled off during the fracturing process, the remaining product phases with bright colors attached to the grain boundaries could still be detected. The typical EDS pattern and main composition of the initial corrosion phase and grain matrix are presented in Figure 7 and Table 3, respectively.

The composition results indicate that the initial corrosion products (Spot 1 in Figure 7) were Fe–As–C-based phases. No obvious O or other elements could be detected. This result further confirms the previous conclusion that C promoted corrosion. However, a relatively high O content was detected in the grain matrix (Spot 2 in Figure 7). The results indicate that C promoted corrosion, particularly during the initial corrosion stage, by forming an Fe–As–C system. As the reduction process temperature was relatively high, O was transferred to the inner matrix before extensive corrosion by elemental As and C.

### 3.4. Analysis of Carbon Sources

**Figure 8:**
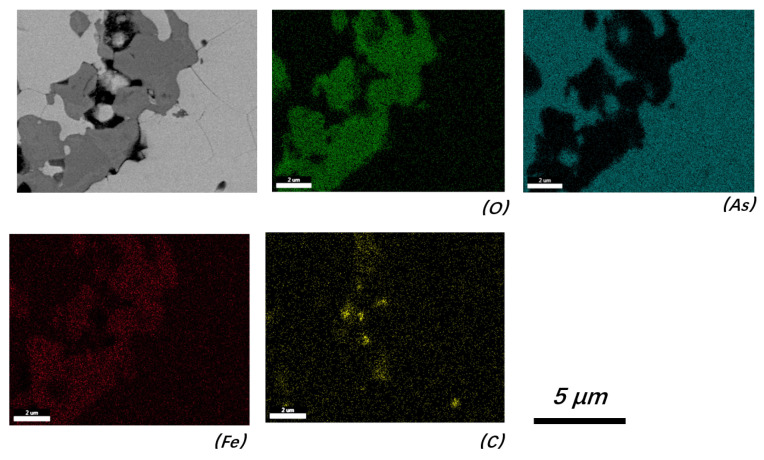
Area scanning of a cross-section near the outer surface of the steel wall (Position 3, backscattered electron image).

EDS area scanning confirmed the existence of C particles with approximate sizes of 0.4–5 μm in the corrosion system. Figure 8 shows a typical cross-section near the outer surface of the steel wall (sampled from Position 3 in Figure 2), where the remaining iron oxide was surrounded by As-based phases, and typical C particles could be observed near the iron-oxide area. The existence of C particles within the corrosion system indicates that, instead of a gaseous C source (CO_2_ or CO), the C source could be a solid-state raw material. As the charcoal used for reduction contacted the steel wall directly, the highest corrosion rate occurred in this zone (Position 2 in Figure 2 and Figure 3).

Based on the corrosion analysis conducted in this study, C can decrease the eutectic temperature of the system and promote the initial corrosion process. Thus, low-purity products, dust, and unspent charcoal from previous batches should be used with caution, as fine C particles could be enriched in these materials and brought out by gases (such as gaseous arsenic trioxide or elemental arsenic) during heating. The C particles detected within the corroded sample from Position 3 prove this conclusion (no obvious C particles were detected in the initial As_2_O_3_).

## 4. Conclusions

In this study, experiments were conducted to investigate the corrosion process in a steel reactor during elemental As manufacturing. The following conclusions were drawn:

1. The steel walls near the charcoal zone of the reactors exhibited the highest corrosion velocities, with Fe_2_As, FeO, and Fe_3_O_4_ being the main crystal phases detected near the corrosion perforation area.
2. In this study, no evidence related to the effects of Sn, Sb, and Se on the corrosion processes was found. The initial corrosion process was attributed to the formation of an Fe–As–C system at the grain boundaries of the steel matrix.
3. C particles with approximate sizes of 0.4–5 μm were also detected in the corrosion system. These C particles partially originated from reused materials in previous production batches.
4. Owing to the high processing temperature, O was transferred to the inner matrix before extensive corrosion by elemental As and C.

In this study, we discovered that C particles from initial or reused raw materials promote the initial corrosion of steel reactors by the formation of an Fe–As–C system at the grain boundaries of the steel matrix, which suggests that a standard on fragmentation of carbonaceous materials during the high-temperature reduction and content limitation of C within reused materials is required. Based on the results, an inert atmosphere in the induction furnace is also recommended. Moreover, as few studies have been reported on the thermodynamics and kinetics information of the corrosion process, especially for essential data on Fe–As–C-based systems, which are lacking, related studies will be conducted in the future.

## Figures and Tables

**Table 1:** Composition of steel reactor (wt%).

C	Mn	Si	P	S	Fe
0.18	1.27	0.36	0.008	0.003	rest

**Table 2:** Typical composition of corroded samples (wt%).

Position	Fe_2_O_3_	As_2_O_3_	MnO	SiO_2_	CaO	SO_3_	SeO_2_
2	73.91	23.75	0.61	1.02	0.0525	0.0803	0.0158
1	90.15	6.44	0.60	1.52	0.2821	0.1227	0.0131

(Given as oxides; C is not included).

**Table 3:** Composition of EDS measurements (wt%).

Position	Fe	As	C	O
Spot 1	41.96	53.88	3.96	-
Spot 2	90.75	2.55	2.54	4.15

## Data Availability

The original contributions presented in this study are included in the article. Further inquiries can be directed to the corresponding authors.

## Abbreviations

The following abbreviations are used in this manuscript:

SEM	Scanning electron microscopy
EDS	Energy-dispersive spectroscopy
XRD	X-ray diffraction
XRF	X-ray fluorescence
